# Role of Polycomb Group Protein Cbx2/M33 in Meiosis Onset and Maintenance of Chromosome Stability in the Mammalian Germline

**DOI:** 10.3390/genes2010059

**Published:** 2011-01-11

**Authors:** Claudia Baumann, Rabindranath De La Fuente

**Affiliations:** 1 Female Germ Cell Biology Group, Department of Clinical Studies, Center for Animal Transgenesis and Germ Cell Research, School of Veterinary Medicine, University of Pennsylvania, New Bolton Center, 382 West Street Road, Kennett Square, PA 19348, USA; E-Mail: cbaumann@uga.edu; 2 Department of Physiology and Pharmacology, College of Veterinary Medicine, University of Georgia, 501 D.W. Brooks Drive, Athens, GA 30602, USA

**Keywords:** oogenesis, pericentric heterochromatin, epigenetic modifications, chromatin remodeling, retinoic acid, sex determination

## Abstract

Polycomb group proteins (PcG) are major epigenetic regulators, essential for establishing heritable expression patterns of developmental control genes. The mouse PcG family member M33/Cbx2 (Chromobox homolog protein 2) is a component of the Polycomb-Repressive Complex 1 (PRC1). Targeted deletion of *Cbx2/M33* in mice results in homeotic transformations of the axial skeleton, growth retardation and male-to-female sex reversal. In this study, we tested whether *Cbx2* is involved in the control of chromatin remodeling processes during meiosis. Our analysis revealed sex reversal in 28.6% of XY^−/−^ embryos, in which a hypoplastic testis and a contralateral ovary were observed in close proximity to the kidney, while the remaining male mutant fetuses exhibited bilateral testicular hypoplasia. Notably, germ cells recovered from *Cbx2*^(XY−/−)^ testes on day 18.5 of fetal development exhibited premature meiosis onset with synaptonemal complex formation suggesting a role for Cbx2 in the control of meiotic entry in male germ cells. Mutant females exhibited small ovaries with significant germ cell loss and a high proportion of oocytes with abnormal synapsis and non-homologous interactions at the pachytene stage as well as formation of univalents at diplotene. These defects were associated with failure to resolve DNA double strand breaks marked by persistent γH2AX and Rad51 foci at the late pachytene stage. Importantly, two factors required for meiotic silencing of asynapsed chromatin, ubiquitinated histone H2A (ubH2A) and the chromatin remodeling protein BRCA1, co-localized with fully synapsed chromosome axes in the majority of *Cbx2*^(−/−)^ oocytes. These results provide novel evidence that Cbx2 plays a critical and previously unrecognized role in germ cell viability, meiosis onset and homologous chromosome synapsis in the mammalian germline.

## 1. Introduction

In all sexually reproducing organisms, germ cells have the monumental task of transmitting genetic information through subsequent generations. Primordial germ cells (PGCs) must undergo epigenetic reprogramming, meiotic recombination and two subsequent chromosomal divisions in order to give rise to mature haploid sperm or eggs. Following colonization of the genital ridge (day 12.5 post coitum in mice), PGCs undergo two or three mitotic divisions before proceeding into a final round of DNA replication and entering a pre-meiotic stage [1,2]. In fetal female germ cells, the transition from mitosis to meiosis ensues approximately on day 14.5 post coitum (pc) and is characterized by a retinoic acid-dependent up regulation of meiosis-specific genes, including the synaptonemal complex protein 3 (SYCP3), followed by striking changes in chromosome configuration. In direct contrast, meiosis is not initiated in the male germline until shortly before puberty. This meiotic arrest in the male is mediated by secretion of the retinoid metabolizing enzyme CYP26B1 from Sertoli cells which promotes degradation of retinoic acid (RA) produced by the mesonephros [1,3,4].

Although it is well established that RA of somatic origin is the predominant metabolic influence in triggering meiosis onset, germ cell-intrinsic factors such as the RNA-binding protein DAZL are also critical for the transition from mitosis to meiosis by promoting transcriptional activation of *Stra8* (stimulated by retinoic acid 8) [5]. Importantly, the unique chromatin conformation in mammalian germ cells and/or germline-specific histone posttranslational modifications may also underlie the specific actions of RA in both male and female germ cells [6,7]

Extensive changes in chromatin modifications are critical from the earliest stages of primordial germ cell differentiation. Global genome reprogramming events may also be dependent on signaling molecules from the surrounding somatic cells of the embryonic gonad [8–11]. Chromatin remodeling in the germline is essential to modulate chromosome structure and, hence, for the establishment of proper homologous chromosome synapsis [12–14]. Notably, biochemical analyses as well as several genetic mouse models support the existence of critical factors that might be exclusively involved in the control of germline-specific epigenetic modifications essential for the successful completion of meiotic prophase I in the female as well as the male germline [15,16]. However, the role of epigenetic modifications in meiosis onset is less clear.

The Polycomb group (PcG) proteins are a family of transcriptional repressors that form large multi-protein complexes, which exert their function through the modulation of higher order chromatin structure [17]. The mouse Polycomb protein M33 and its human homolog CBX2 are members of the Polycomb repressive complex 1 (PRC1). Mutations in the *Cbx2*/*M33* gene induce a range of congenital birth defects such as transformations of the axial skeleton associated with premature homeotic (*Hox*) gene expression [18,19], in addition to severe abnormalities in sexual development including male to female sex reversal in humans [20] and mice [18,21]. Despite the impact of *CBX2* mutations on human health, little is known regarding the molecular and/or epigenetic factors that predispose the mammalian gonad to abnormalities of sexual differentiation. Moreover, the potential role of *Cbx2*/*M33* in the modulation of large-scale chromatin structure during meiosis remains largely unexplored. In this study, we show that functional ablation of Cbx2 protein results in precocious meiotic onset in fetal male germ cells as well as a spectrum of meiotic defects in female germ cells. Our results support an essential role for Cbx2 in germ cell development as well as the establishment of homologous chromosome synapsis during meiosis. Importantly, the meiotic phenotype observed in mutant fetal testicular germ cells indicates that Cbx2 plays a critical role in antagonizing the effects of retinoic acid on meiotic onset and is required for maintenance of the pre-meiotic arrest of fetal male germ cells in mammals.

## 2. Results and Discussion

### 2.1. Loss of Cbx2 Function Causes Gonadal Growth Retardation and Male-to-Female Sex Reversal on a BALB/C Genetic Background

Male-to-female sex reversal has been previously reported in a *Cbx2* mutant mouse model established on the C57BL/6Njcl (B6) genetic background [21]. These mice carry an insertion of a neomycin cassette in exon 5 of the *Cbx2* coding sequence resulting in deletion of the *C*-terminal portion of the Cbx2 protein. Although expected Mendelian inheritance patterns were observed at birth, postnatal lethality affected up to 60% of the mutant offspring before 5–6 weeks of age. In this strain, all surviving chromosomally male *Cbx2*^(XY−/−)^ mice exhibited male to female sex reversal with female or intersex-type external genitalia. In addition, the internal reproductive organs consisted of bilateral ovaries containing follicles in 50% of males as well as the presence of both an ovary and a contralateral testis in 25% of mutant males [21].

Abnormalities of sex differentiation have also been reported in an alternative *Cbx2* knockout mouse model generated on a BALB/C genetic background [18,21]. However, the extent of phenotypic sex reversal observed in this model is not known. Previous studies have documented striking differences in the level of sensitivity to phenotypic sex reversal amongst different mouse strains and genetic backgrounds [22]. Therefore, we set out to determine the type and severity of gonadal defects observed in BALB/C *Cbx2*^(−/−)^ knockout mice. This model [18] involved the targeted deletion of the 5′ untranslated region including the translation start site and the first four exons of the *Cbx2* coding sequence leading to a null mutation of the Cbx2 protein. The majority of *Cbx2*^(−/−)^ mice in this model show neonatal lethality within hours after birth likely due to proliferative defects in several cell types and homeotic transformations [23].

**Figure 1:**
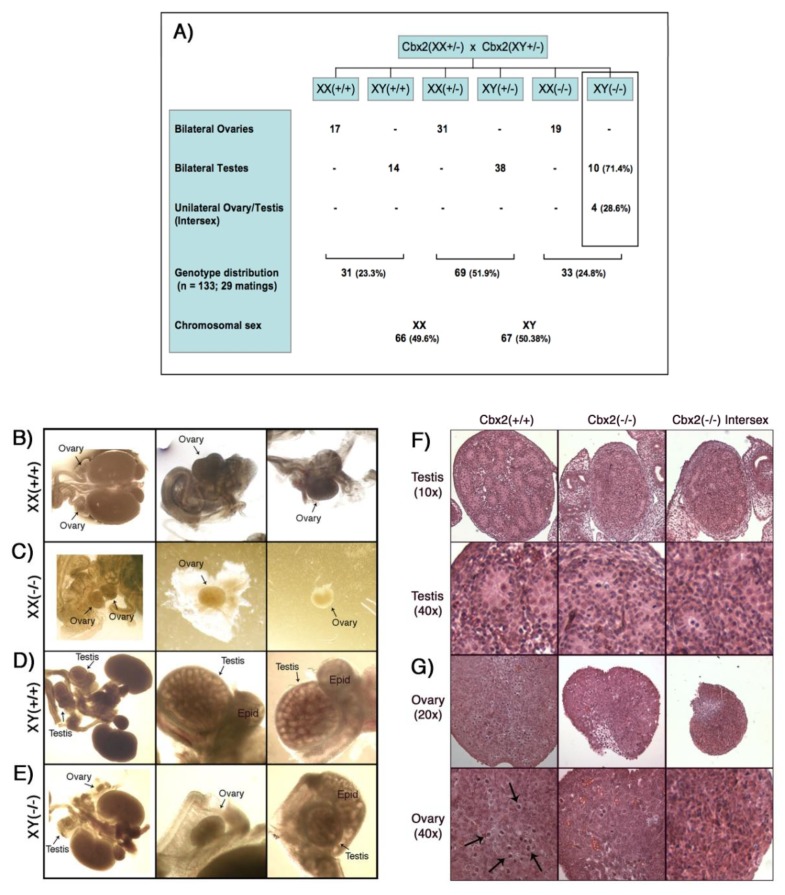
Targeted deletion of *Cbx2* leads to sex reversal and gonadal hypoplasia. (**A**) Correlation between genotype, phenotype and chromosomal sex in fetal offspring of heterozygous (*Cbx2*^(+/−)^ intercrosses; (**B**) Gonadal differentiation in a female wild type fetus on day 18.5 pc; (**C**) Stage-matched *Cbx2*^(XX−/−)^ mutant fetus showing ovarian hypoplasia; (**D**) Anatomic disposition and testicular morphology in a male *Cbx2*^(XY+/+)^ control fetus with prominent testis cord development; (**E**) Sex reversal in a *Cbx2*^(XY−/−)^ male containing both a small ovary as well as a contralateral small testis; (**F**) Wild type fetuses exhibit well-developed testicular cords. In contrast, mutant *Cbx2*^(XY−/−)^ fetuses with bilateral testicular hypoplasia as well as intersex embryos show poor definition of tubular structures; (**G**) Ovarian histology in control ovaries reveals numerous oocytes in meiosis (arrows). In contrast, mutant gonads show reduced meiotic germ cell numbers and hypoplasia.

Mice heterozygous for *Cbx2* (Cbx2^(+/−)^are viable and fertile and fetuses recovered on day 18.5 post coitum from heterozygous matings revealed a normal ratio of *Cbx2*^(+/+)^/*Cbx2*^(+/−)^/*Cbx2*^(−/−)^ genotypes of 31:69:33 (Figure 1A). The chromosomal sex of all 18.5 day post coitum (dpc) fetuses was determined by the presence or absence of *Sry* gene sequences as detected by PCR. Chromosomally female fetuses from all genotypes (*n* = 66) contained bilateral ovaries (Figure 1 A–C). However, *Cbx2*^(XX−/−)^ females exhibited small ovaries (Figure 1C) compared to control *Cbx2*^(XX+/+)^ females (Figure 1B). Importantly, in contrast to wild type controls (Figure 1D; *n* = 14) chromosomally male *Cbx2*^(XY−/−)^ mutant fetuses (*n* = 14) exhibited severe testicular growth retardation accompanied by impaired formation of testicular cords and in extreme cases unilateral sex reversal as reflected by the presence of a hypoplastic testis and a contralateral ovary in 28.6% of *Cbx2*^(XY−/−)^ fetuses (Figure 1E). As expected, the reproductive tracts obtained from wild type controls (Figure 1D) always exhibited bilateral testes at a caudal position towards the inguinal region. However, although the size and morphological appearance of the epididymis was similar to wild type controls, both the testis and adjacent epididymis remained in close proximity to the kidneys in *Cbx2*^(XY−/−)^ mutant fetuses (Figure 1E). Histological examination on day 18.5 pc confirmed a reduction in the size and number of testicular cords in *Cbx2*^(XY−/−)^ mutant males presenting bilateral testes as well as in the testis of mutant males showing unilateral sex reversal compared to wild type controls *Cbx2*^(XY+/+)^ (Figure 1F). Growth retardation and germ cell loss was apparent in both, testes (Figure 1F) as well as ovaries (Figure 1G) from *Cbx2*^(XY−/−)^ mutants compared to wild type gonads. Similarly, histological sections obtained from *Cbx2*^(XX−/−)^ females confirmed the presence of small ovaries compared to controls (Figure 1G).

These results indicate that, in addition to its role in sex determination, *Cbx2* plays an important role in germ cell viability during fetal development. Importantly, our study reveals major differences in the sex reversal phenotype of *Cbx2* knockout mice generated on the BALB/C genetic background [18] compared to the phenotype described in *Cbx2* deficient mice on the C57BL/6Njcl (B6) background [21]. For example, the majority of *Cbx2* null mice on the B6 background exhibit male to female sex reversal and approximately 50% of surviving adult mice exhibited bilateral ovaries. However, none of the knockout males analyzed contained bilateral testes [21]. In contrast, our study reveals that in the BALB/C model <30% of mice exhibit sex reversal. This is consistent with previous studies indicating an extreme sensitivity of the B6 background to genetic perturbations of the sex determination pathway due to higher levels of expression for several genes associated with female gonadal differentiation [22,24]. Interestingly, the majority of affected males in the BALB/C model exhibit bilateral testis and mutant females presented fully differentiated ovaries, making this strain uniquely suited for the analysis of the functional ablation of Cbx2 in mammalian germ cells during the critical window of epigenetic reprogramming and the mitotic to meiotic transition in the germline. These results provide the first evidence indicating a role for Cbx2 in germ cell development.

### 2.2. Premature Meiosis Onset in Fetal Cbx2^(XY−/−)^ Testicular Germ Cells

**Figure 2:**
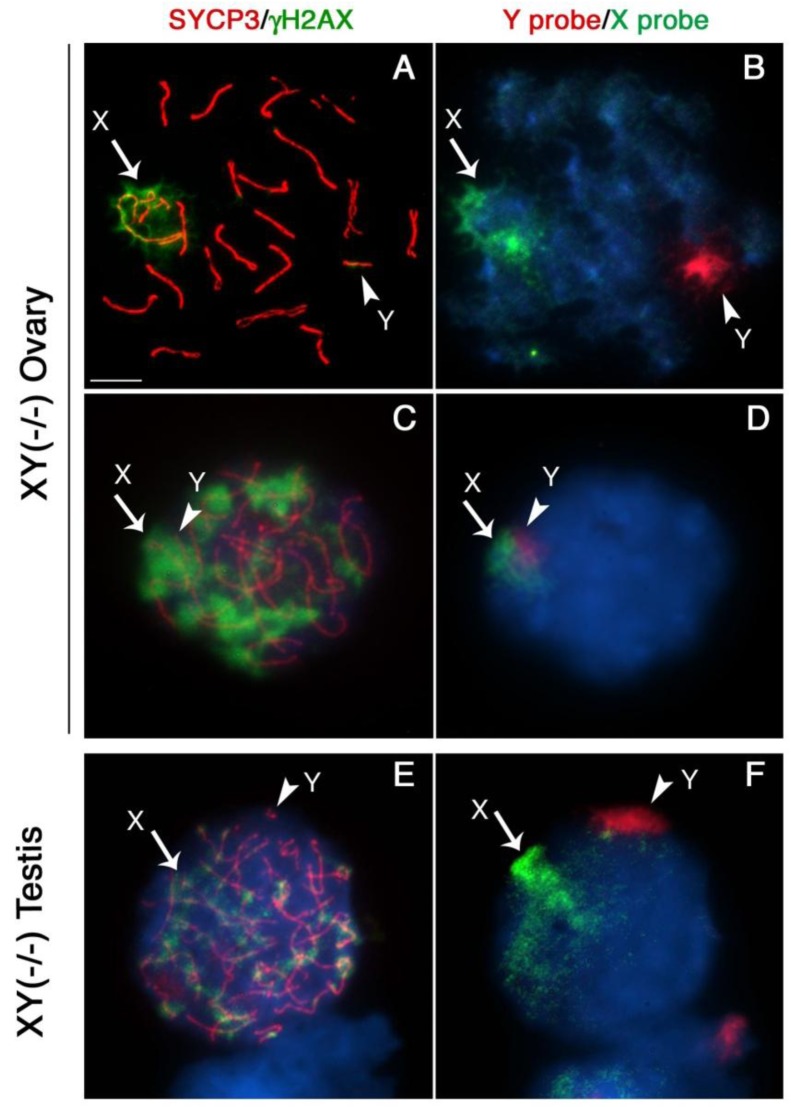
Precocious meiosis onset in *Cbx2*^(XY−/−)^ fetal gonads. (**A**) *Cbx2*^(XY−/−)^ oocyte recovered from an ovary of a unilaterally sex reversed embryo showing completely asynapsed sex chromosomes and persistent γH2AX staining associated with the X chromosome; (**B**) The position of the X (green, arrow) and the Y (red, arrowhead) chromosome is shown; (**C**, **D**) Partial synapsis of the sex chromosomes associated with abundant γH2AX signals (green) at sex chromatin and autosomes in a *Cbx2*^(XY−/−)^ oocyte at the late zygotene-early pachytene stage; (**E**, **F**) Evidence for precocious onset of meiotic prophase I in fetal testicular germ cells obtained from a *Cbx2*^(XY−/−)^ embryo with bilateral testes. Synaptonemal complexes at meiotic chromosome cores are labeled with SYCP3 (red) and γH2AX staining (green). DNA is shown in blue. Scale bar = 10 μm.

Next, we determined whether lack of Cbx2 function in the mammalian germline is associated with abnormal chromatin-related events during meiosis. Analysis of meiotic configurations on surface spread oocytes obtained on day 18.5 pc from the ovaries of *Cbx2*^(XY−/−)^ males exhibiting unilateral sex reversal revealed abnormal homologous chromosome synapsis involving one or two bivalents as well as several chromosome fragments as determined by synaptonemal complex staining with an anti-SYCP3 antibody (Figure 2A; red). Notably, phosphorylated histone H2AX (γ-H2AX) remained associated with the asynapsed X chromosome and, to a lesser extent, with the Y chromosome (Figure 2A; green). Immuno-FISH experiments using sex chromosome-specific paint probes revealed that in >80% of *Cbx2*^(XY−/−)^ oocytes the X (green, arrow) and Y (red, arrowhead) chromosomes fail to undergo partial synapsis (Figure 2B). Moreover, although γH2AX was found associated with the sex chromosomes in the remaining mutant oocytes that exhibited sex chromosome pairing, a typical XY body was not detectable (Figure 2C, D). These results indicate that XY oocytes from sex reversed *Cbx2* null gonads exhibit meiotic abnormalities in the form of chromosome breaks in addition to the lack of synapsis of the sex chromosomes.

Surprisingly, our studies also revealed that germ cells obtained from the contralateral testis of *Cbx2*^(XY−/−)^ male fetuses on day 18.5 pc undergo precocious entry into meiosis demonstrated by *bona fide* synaptonemal complex formation and a chromosome configuration consistent with the zygotene stage of meiosis with asynapsed chromosomes and diffuse γH2AX staining (not shown). Strikingly, premature meiotic germ cells were also present in *Cbx2*^(XY−/−)^ mutant males with bilateral testes, albeit at less advanced stages of prophase I compared to XY ovarian oocytes (Figure 2E, F). These results indicate that Cbx2 plays a critical role in the pathway responsible for the pre-meiotic arrest in the mammalian male germline. Abnormal chromosome synapsis in *Cbx2*^(XY−/−)^ mutants suggests, moreover, a role for Cbx2 in meiotic chromosome synapsis in the male germline. However, whether chromosome synapsis and XY body formation are affected in Cbx2^(XY−/−)^ spermatocytes undergoing precocious meiosis remains to be investigated.

Our results are consistent with previous studies on the meiotic prophase configuration in XY oocytes obtained from the XY^tdym1^ mouse strain, in which only 19% of oocytes exhibit partial synapsis of the sex chromosomes with no apparent sex body formation. Interestingly, in this model, histone posttranslational modifications such as ubiquitinated H2A remained associated with the X chromosome in 50% of oocytes [25]. However, the functional significance of these histone modifications for transcriptional silencing of the sex chromosomes in XY oocytes requires further investigation.

Notably, the identification of fetal male germ cells with precocious synaptonemal complex formation and a zygotene-like configuration on day 18.5 pc constitutes the first evidence indicating that Cbx2 may play a critical role in maintenance of the pre-meiotic arrest in fetal male germ cells. Previous studies elegantly demonstrated that treatment of embryonic testes with retinoic acid agonists or inhibitors of the RA metabolizing enzyme CYP26 induce premature *Stra8* expression in cultured male gonads [3,4]. In addition, treatment of fetal testis with the histone deacetylase inhibitor TSA induced *Stra8* expression and SYCP3-positive staining on histological sections [7]. However, the presence of meiotic figures in these experimental systems remained to be demonstrated. Our results show for the first time that in the absence of Cbx2 function, fetal testicular germ cells prematurely transit into meiosis and exhibit synaptonemal complex formation, albeit at a lower frequency compared to ovarian germ cells. Results obtained following exposure of fetal testes to histone deacetylase inhibitors suggest that, in addition to the stimulus provided by RA, epigenetic mechanisms might also contribute to prevent premature meiosis onset in the male germline [7]. Interestingly, valproic acid, a specific inhibitor of histone deacetylases, has been recently shown to reduce the expression of *Cbx2* transcripts in mouse embryos on day 8 pc [26]. This suggests that the role of Cbx2 in modulating large-scale chromatin structure may be critical for antagonizing the potential effects of retinoic acid exposure on premature meiotic entry in the male germline. In support of this notion, previous studies indicate that Cbx2 is essential to control the developmental window of responsiveness to retinoic acid during fetal development [19].

### 2.3. Loss of Cbx2 Results in Abnormal Chromosome Synapsis and Structural Damage of Meiotic Chromosomes in Female Germ Cells

**Figure 3:**
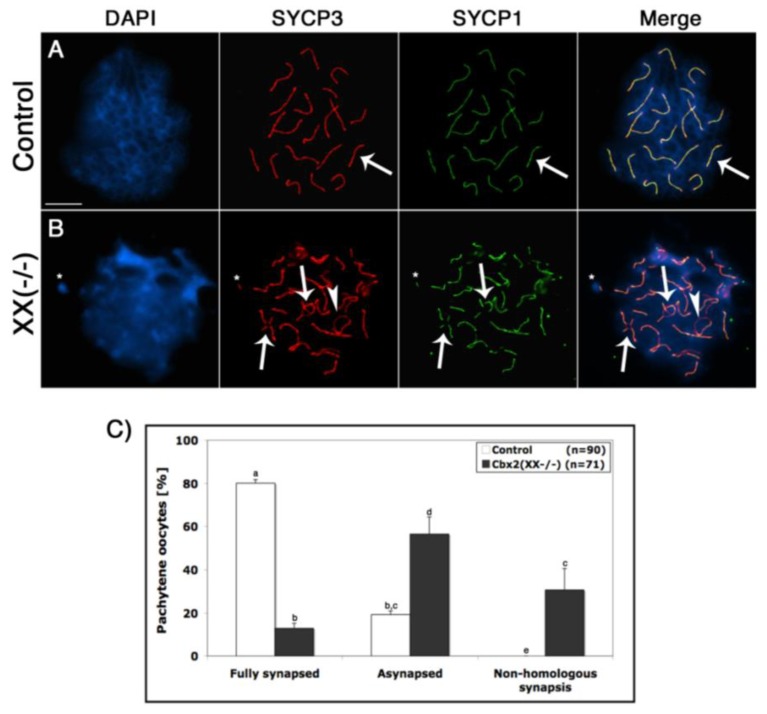
Abnormal synapsis and non-homologous chromosome interactions in *Cbx2*^(XX−/−)^ mutant oocytes. (**A**) Chromosome synapsis (arrow) in control oocytes at the pachytene stage showing complete co-localization of lateral (SYCP3, red) and central (SYCP1, green) elements of the synaptonemal complex; (**B**) Stage-matched *Cbx2*^(XX−/−)^ oocyte presenting abnormal synapsis (arrowhead), non-homologous chromosome interactions between two partially synapsed bivalents (arrows) as well as structural damage in the form of prominent chromosome breaks (asterisk). DNA is shown in blue; (**C**) Proportions of pachytene stage oocytes obtained from control and mutant fetuses on day 18.5 pc showing incomplete chromosome synapsis and non-homologous interactions. Scale bar = 10 μm.

Cbx2 associates with pericentric heterochromatin domains during mitosis in both murine somatic and embryonic stem cells. The protein also localizes to facultative heterochromatin at the inactive X chromosome suggesting a potential role in heterochromatin formation [27–29]. However, whether Polycomb group proteins, such as Cbx2, play a role in meiotic chromosome synapsis in the female germline is not known. Therefore, we undertook a detailed analysis of meiotic synapsis and recombination events in Cbx2 mutant oocytes. The dynamics of chromosome synapsis was determined following simultaneous staining of the lateral elements with SYCP3 (red) as well as the central elements of the synaptonemal complex with SYCP1 (green). At the pachytene stage, 80% of wild type oocytes (*n* = 90) exhibited fully synapsed chromosome bivalents (Figure 3A arrow, C). In contrast, mutant oocytes (*n* = 71) exhibited a significant increase in the incidence (57%) of abnormal synapsis (Figure 3B, arrowhead) and Figure 3C. Moreover, chromosomal associations between two partially synapsed bivalents, indicative of non-homologous chromosome interactions (arrows), were commonly observed in >30% of Cbx2^(XX−/−)^ oocytes (Figure 3B, C). These results indicate a previously unrecognized role of Cbx2 in chromosome pairing and homologous chromosome synapsis in female germ cells.

#### 2.3.1. Persistence of DNA Double Strand Breaks and Early Recombination Intermediates at the Pachytene Stage in Cbx2^(XX−/−)^ Oocytes

**Figure 4:**
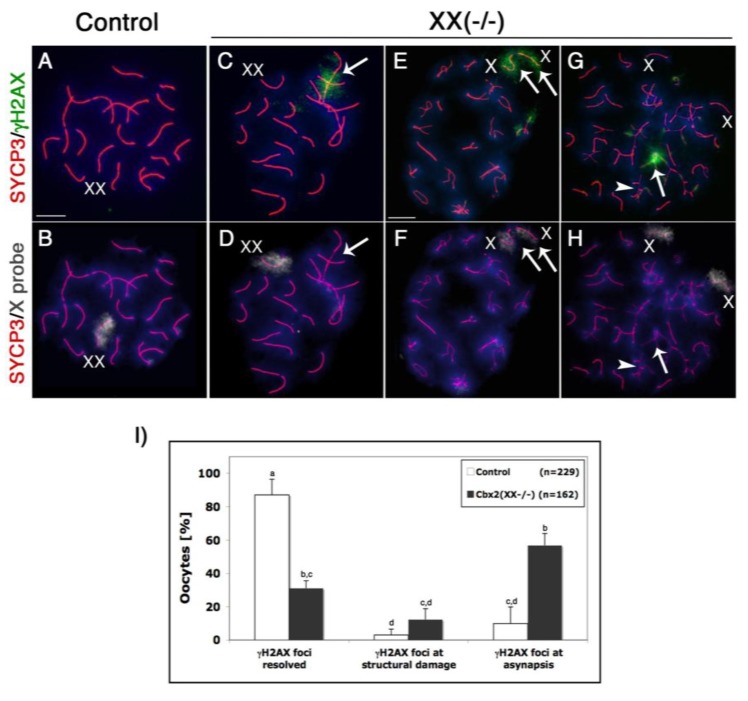
Persistence of H2AX phosphorylation and presence of univalent chromosomes in *Cbx2*^(XX−/−)^ oocytes. (**A**) Late pachytene oocyte obtained from a control *Cbx2*^(XX+/+)^ ovary on day 18.5 pc exhibiting complete chromosome synapsis (20 bivalents). Note the absence of γH2AX staining; (**B**) The position of the X chromosome bivalent (white) is indicated; (**C**, **D**) Mutant oocyte exhibiting a chromosome bivalent with axial gaps in the synaptonemal complex decorated by γH2AX staining (green) suggesting the presence of structural chromosome damage (arrow); (**E**, **F**) Persistence of H2AX phosphorylation (green) in *Cbx2*^(XX−/−)^ oocytes showing failure to resolve DSBs at X chromosome univalents (arrows); (**G**, **H**) *Cbx2*^(XX−/−)^ oocyte at diplotene stage exhibiting γH2AX staining at asynapsed chromosomes (arrow). Note the presence of X chromosome univalents and non-homologous associations lacking γH2AX foci (arrowhead). Synaptonemal complexes are labeled with SYCP3 (red) and DNA is shown in blue. The position of the X chromosomes is marked with (X); (**I**) Proportion of mutant oocytes exhibiting abnormal γH2AX patterns. Scale bars = 10 μm.

Initiation of programmed DNA double strand breaks (DSBs) during leptotene stage of meiosis is marked by a striking accumulation of the phosphorylated form of the histone variant H2AX (γH2AX), which is required for the recruitment of factors involved in the repair of DSBs during chromosome synapsis [30–32]. Following resolution of DSBs at the pachytene stage, persistent γH2AX marks the majority of asynapsed chromosomes or chromosome segments and its presence in fully synapsed bivalents has been associated with structural chromosome damage [33,34]. Therefore, we set out to determine the patterns of γH2AX nuclear localization in Cbx2 mutant oocytes at the pachytene and diplotene stage. Our analysis revealed that γH2AX was undetectable in fully synapsed chromosomes in the majority (87%) of control oocytes (*n* = 229) at the pachytene stage (Figure 4A, B, I). In contrast, a spectrum of meiotic abnormalities was detected in *Cbx2*^(XX−/−)^ mutant oocytes (*n* = 162). For example, γH2AX (green) was observed in chromosome bivalents showing full synapsis and/or axial gaps in the synaptonemal complex (red) in 12.3% (*p*< 0.05) of mutant germ cells (Figure 4C, D and I) consistent with the presence of structural chromosome damage. Moreover, pronounced γH2AX signals were also detected at asynapsed chromosomes in 56.7% (*p*< 0.05) of mutant oocytes (Figure 4I). This observation was further substantiated by X chromosome-specific FISH analyses indicating the persistence of γH2AX at X chromosome univalents in a subpopulation of Cbx2 deficient oocytes at the diplotene stage (Figure 4E, F). However, we also found evidence for the presence of X chromosome univalents with no γH2AX staining suggesting that these chromosomes failed to remain bound by chiasmata at the diplotene stage following initial synapsis and resolution of DSBs at the pachytene stage (Figure 4G, H).

**Figure 5:**
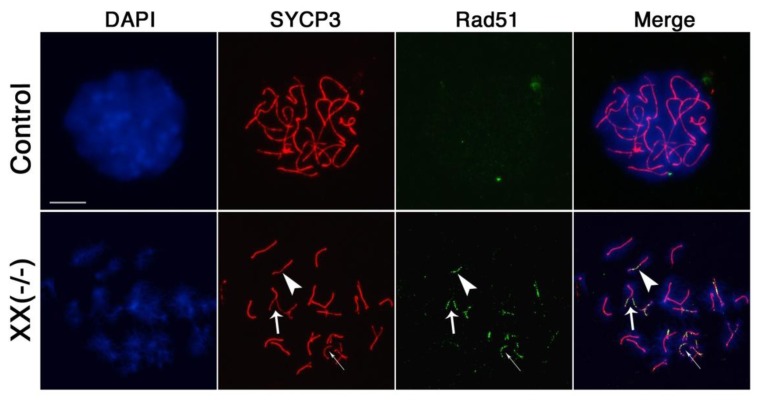
Rad51 foci associate with asynapsed chromosomes and chromosome segments in *Cbx2* mutant oocytes. Complete meiotic chromosome synapsis in control oocytes is associated with the timely resolution of DSBs and lack of Rad51 foci at the pachytene stage (upper panel). In *Cbx2*^(XX−/−)^ mutant oocytes non-homologous and incomplete chromosome synapsis leads to persistence of Rad51 foci (green, arrow, thin arrow) on meiotic chromosome axes (lower panel). Moreover, Rad51 foci are also retained at chromosome cores that appear fully synapsed but exhibit axial gaps on the synaptonemal complex (arrowhead). SYCP3 is shown in red and DNA is shown in blue. Scale bar = 10 μm.

The recombination intermediate Rad51 is a *bona fide* marker of DSB formation at recombination nodules in meiotic chromosomes during the zygotene stage. However, Rad51 becomes progressively dissociated from crossover sites following resolution of DSBs and homologous chromosome synapsis at the pachytene stage [35,36]. As expected, Rad51 foci were undetectable in the majority of wild type oocytes showing full synapsis of homologous chromosomes at the pachytene stage (*n* = 101), suggesting a timely repair of DSBs (Figure 5, upper panel). In contrast, >56% of mutant oocytes (*n*= 139) presented Rad51 foci (green) associated with asynapsed chromosomes (Figure 5, lower panel, thin arrow). Notably, >41% of these mutant oocytes showed additional persistent Rad51 foci at synapsed bivalents exhibiting axial gaps in the synaptonemal complex (arrowhead) as well as segments of incomplete synapsis (bold arrow) demonstrating that loss of Cbx2 function interferes not only with proper homologous chromosome synapsis but also with the timely resolution of DSBs.

#### 2.3.2. Activation of the Meiotic Silencing of Unpaired Chromatin (MSUC) Pathway in Cbx2^(XX−/−)^ Oocytes

**Figure 6:**
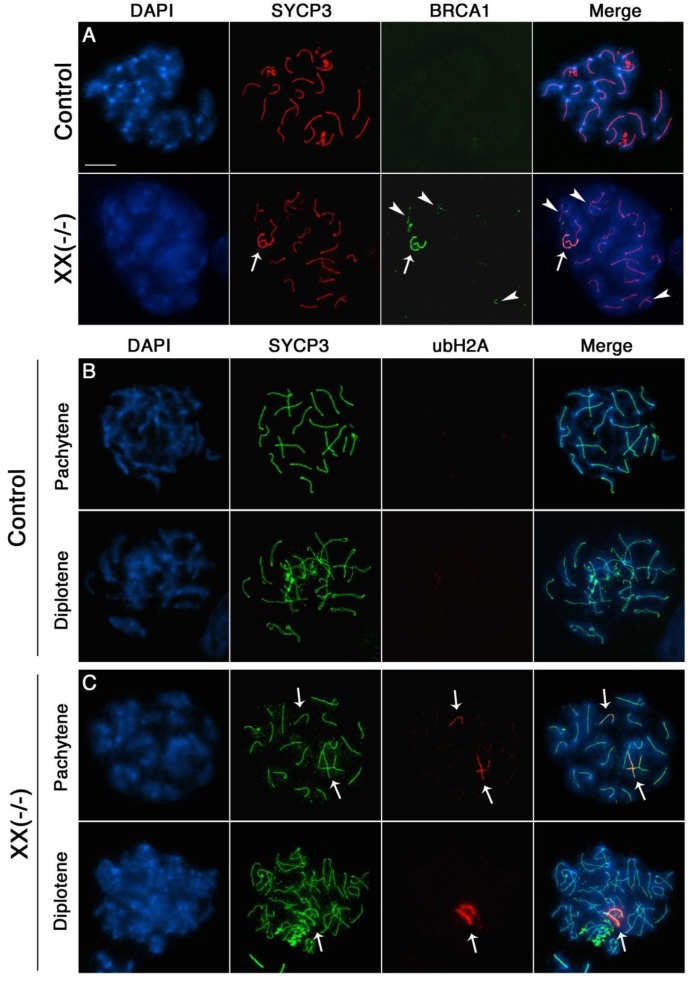
Activation of meiotic silencing of unsynapsed chromatin (MSUC) in *Cbx2*^(XX−/−)^ oocytes. (**A**) Compared to oocytes isolated from control ovaries (upper panel), *Cbx2*^(XX−/−)^ oocytes show prominent BRCA1 labeling (green) at a subset of synapsed chromosome cores (red, arrow), while the majority of incompletely synapsed bivalents show BRCA1 with a focal distribution (arrowheads); (**B**) The histone modification ubH2A (red) is not detectable in control oocytes at the pachytene (upper panel) or the diplotene (lower panel) stage of meiosis; (**C**) In *Cbx2*^(XX−/−)^ mutant oocytes, ubH2A associates with the chromosome cores (green) of asynapsed meiotic bivalents at the pachytene stage (arrows, upper panel) and persists at these chromosomal domains until the diplotene stage (arrow, lower panel). DNA is shown in blue. Scale bars = 10 μm.

**Supplemental Figure S1:**
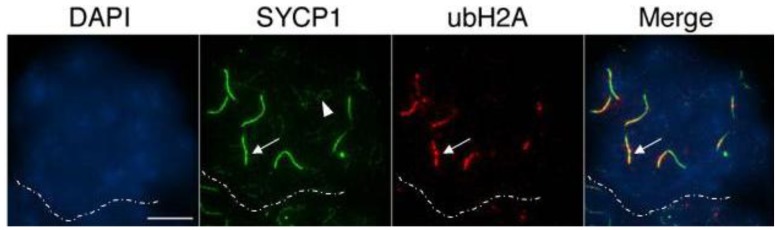
Co-localization of ubH2A (red, arrow) with fully synapsed chromosome bivalents (SYCP1, green) in *Cbx2*^(XX−/−)^ oocytes at the diplotene stage. Arrowhead points to faintly labeled desynapsed bivalent. Scale bar = 10μm.

**Supplemental Figure S2:**
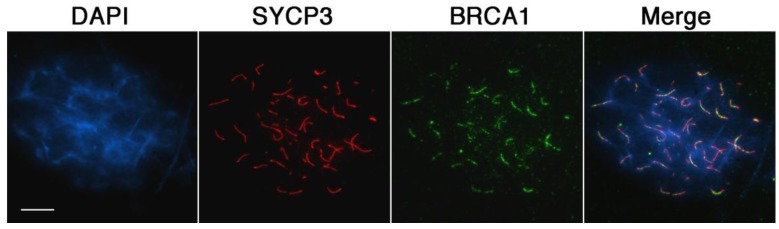
Attenuation of the MSUC response (BRCA1, green) in *Cbx2*^(XX−/−)^ oocytes exhibiting excessive asynapsis (SYCP3, red). Scale bar = 10μm.

The presence of unrepaired DSBs associated with incomplete chromosome synapsis signals the activation of a specialized transcriptional silencing pathway. Meiotic silencing of unsynapsed chromatin (MSUC) is an essential mechanism that recruits the tumor suppressor protein BRCA1 and the histone variant ubiquitinated H2A (ubH2A) to sites of abnormal chromosome synapsis in both female and male germ cells [25,37]. Intriguingly, recent studies have suggested that members of the Polycomb repressive complex 1 (PRC1) as well as the E3 ubiquitin ligases RNF8/RNF168 are required for monoubiquitination of H2A at lysine 119 in somatic cells [38–45]. In order to address whether MSUC is properly initiated in oocytes lacking Cbx2 function, we assessed the sub-nuclear localization patterns of BRCA1 (green) and ubH2A (red) in *Cbx2*^(XX−/−)^ mutant oocytes. While BRCA1 was undetectable in fully synapsed chromosomes of oocytes recovered from control fetuses (Figure 6A, upper panel), *Cbx2*^(XX−/−)^ mutant oocytes showed prominent associations of BRCA1 in one or two fully synapsed bivalents (arrow). In contrast, BRCA1 was also present as small foci on several incompletely synapsed chromosomes (arrowhead) indicating activation of the MSUC response pathway. Similarly, no ubH2A labeling (red) was observed in fully synapsed bivalents of control oocytes at the pachytene or diplotene stage (Figure 6B). In striking contrast, ubH2A (red) was co-localized with asynapsed chromosome axes (green) in the majority of mutant oocytes at the pachytene stage (Figure 6C, upper panel, arrows) and also persisted at fully synapsed chromosome bivalents (Supplemental Figure S1) as well as desynapsed chromosome cores during diplotene stage (Figure 6C, lower panel, arrow). These results indicate that loss of Cbx2 function in the female germline does not preclude activation of the cellular MSUC response. However, evidence suggests that extensive chromosome asynapsis in a subset of mutant oocytes results in focal BRCA1 staining instead of a uniform accumulation of BRCA1 throughout the length of the meiotic chromosome core indicative of an impaired activation of the MSUC response (Supplemental Figure S2), which is in agreement with recent studies indicating that excessive asynapsis depletes the available BRCA1 protein pool leading to an attenuation of the MSUC response [30,46].

Importantly, together with the patterns of Rad51 localization, our findings indicate that failure to repair DSBs results in the persistence of BRCA1 and ubH2A staining on fully synapsed chromosomes at the diplotene stage. Therefore the association of these markers with fully synapsed chromosomes in this model might be a reflection of structural damage to meiotic chromosomes or alternatively, to the lack of establishment of an intimate synapsis between homologous chromosomes. The patterns of ubH2A localization observed at the pachytene and diplotene stage (Figure 6C, lower panel and Supplemental Figure S1) also indicates that component molecules of the PRC1 complex other than Cbx2 are involved in establishing histone H2A ubiquitination in the female germline.

**Figure 7:**
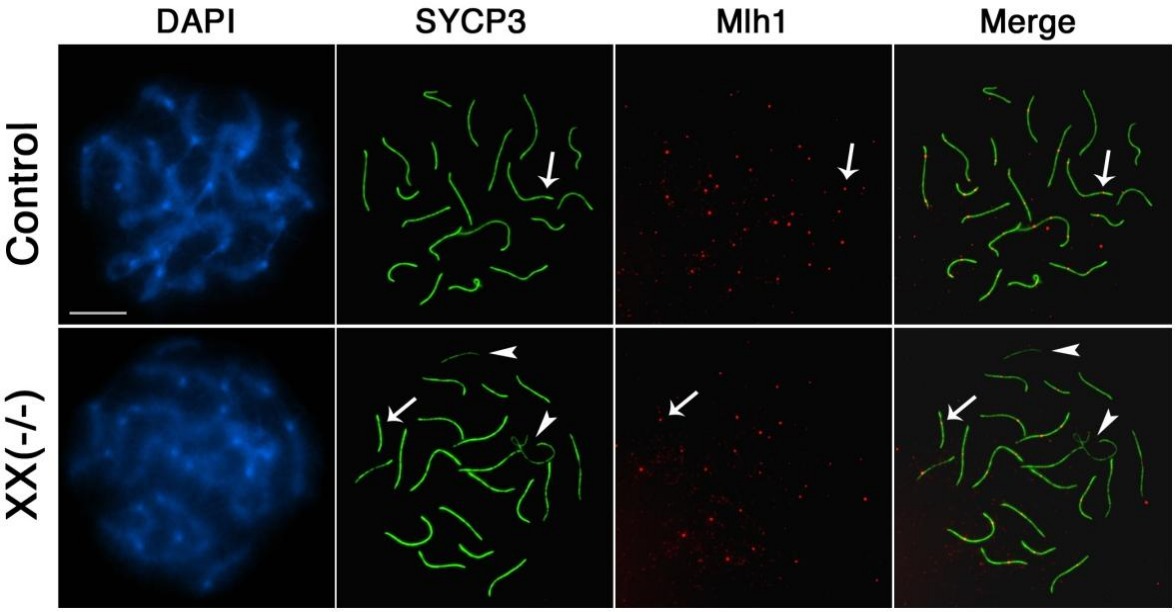
Crossover formation in mutant *Cbx2*^(XX−/−)^ oocytes. Control oocytes (upper panel) as well as oocytes recovered from mutant *Cbx2*^(XX−/−)^ fetuses (lower panel) at 18.5 dpc exhibit 1-2 Mlh1 foci (red) per fully synapsed chromosome bivalent (arrows). In contrast, asynapsed meiotic chromosomes in pachytene stage *Cbx2*^(XX−/−)^ oocytes lack Mlh1 foci due to failure to establish homologous recombination sites (arrowhead). DNA is shown in blue. Scale bars = 10 μm.

Finally, we set out to analyze whether the meiotic defects associated with lack of Cbx2 function interferes with the establishment of mature recombination nodules in mutant oocytes. The mismatch repair protein Mlh1 is a marker of crossover formation and is normally loaded onto mature recombination nodules during mid- to late pachytene stage. Analysis of chromosomal distribution patterns of Mlh1 (red) demonstrated that pachytene oocytes obtained from *Cbx2*^(XX−/−)^ mutant fetuses show, similar to control oocytes (arrow, upper panel), one to two Mlh1 foci on each fully synapsed bivalent (Figure 7, lower panel, arrow). As expected, Mlh1 foci were not detectable at asynapsed chromosome univalents in mutant oocytes due to lack of crossover formation (Figure 7, lower panel, arrowhead). These results suggest that formation of chiasmata proceeds seemingly unimpaired in most but not all fully synapsed homologous chromosomes, while chiasmata formation is precluded in the remaining asynapsed univalents in oocytes lacking Cbx2.

Induction of DSBs is tightly linked to histone H2A phosphorylation (γH2AX) [31,37] as well as to the loading of recombination intermediates such as Rad51, Mre11 [47], Msh2 [48] and Dmc1 [35,49] at early recombination nodules. The patterns of γH2AX and Rad51 staining in *Cbx2*^(XX−/−)^ mutant oocytes indicate that induction of DSBs and loading of recombination intermediates occurs normally in this model. However, failure to resolve DSBs results in recombination errors and structural damage to chromosomes and may interfere with the formation of chiasmata following homologous chromosome synapsis [32,36,47–52]. Therefore, the presence of such meiotic defects may predispose Cbx2 deficient oocytes to the formation of chromosome univalents at the diplotene stage.

Our study has uncovered a novel role for the Polycomb group protein Cbx2 in meiosis through the maintenance of genome stability during this critical developmental window. Yet, precocious meiosis in testicular germ cells from *Cbx2*^(XY−/−)^ mutant fetuses with bilateral testes as well as sex reversed mutants exhibiting a unilateral testis grant unexpected insight into male meiosis onset. Importantly, our results reveal a novel role for Cbx2 protein in meiotic chromosome synapsis in mammalian oocytes.

Little is known regarding the function of Polycomb group proteins in mammalian germ cells. Nevertheless, PcG proteins have been shown to play important roles in the maintenance of chromatin structure and organization during developmental transitions through the establishment of a characteristic and heritable epigenetic landscape. Accordingly, these highly conserved proteins associate in large multi-protein complexes that are capable of altering the nucleosomal composition through the initiation of modifications at specific histone tails, such as H3K27me3 [53,54]. Cbx2 is thought to act as transcriptional regulator at developmental target genes in the context of such complexes [29], although our understanding of the specificity of the particular targeting mechanism is limited. Previous studies have shown that Cbx2 expression, along with other components of PRC1 (Bmi1 and Rae28), is detectable between day 8.5 and 18.5 dpc in whole embryo extracts and is an abundant protein in the adult ovary and testis [55]. Cbx2 is known to localize to facultative heterochromatin of the inactive X chromosome [27,28], to centromeric heterochromatin at metaphase in fibroblasts [29] and to Hoechst-bright chromocenters in embryonic stem cells [28], while it is excluded from the nuclear macrochromatin domain forming the XY body in mouse spermatocytes [56].

PcG interactions with chromatin domains have long been attributed to the chromodomain, a conserved feature common to all Chromobox proteins [57]. However, using bimolecular fluorescence complementation (BiFC) analysis, a recent study indicates that the chromodomain as well as the chromobox of Cbx proteins might be dispensable for DNA binding as such [28]. Interestingly, Cbx2 differs from all other Cbx proteins by a newly identified protein domain, the AT-Hook [58]. AT-Hook motifs are conserved domains found in many DNA-binding proteins and are thought to enhance protein interactions with AT-rich chromatin domains [59]. According to this model, Cbx2 may promote chromatin remodeling directly through interactions with the minor groove of AT-rich satellite DNA sequences, while simultaneous binding of the *N*-terminal Cbx2 chromodomain to specific histone tails (H3K27me3, H3K4me3, H3K9ac) within the adjoining nucleosome could complete the establishment of the remodeled chromatin status [28,58].

Importantly, chromatin remodeling processes are essential for homologous chromosome pairing, synapsis and proper chromosome segregation during meiosis in a wide array of organisms from plants [60] to mammals [15,61–65]. The phenotype observed in fetal oocytes lacking Cbx2 might suggest a functional role for Cbx2 in chromatin remodeling required for the establishment and/or repair of DSBs, homologous chromosome pairing or sister chromatid cohesion between homologous chromosomes. Interestingly, several chromodomain and PcG-associated proteins have previously been demonstrated to play a role in meiotic sister chromatid cohesion in *Drosophila* [66], DSB repair in mammals [67,68] as well as locus-specific imprinting during meiosis in fission yeast [69]. However, further analyses are necessary to identify the precise mechanism of action of Cbx2 in conferring genome stability during meiosis in the female germline.

## 3. Experimental Section

### 3.1. Mice

All animal experiments were approved by the institutional animal use and care committee of the University of Pennsylvania according to National Institutes of Health guidelines.

Mice heterozygous for a targeted deletion of *Cbx2* (C.129P2-Cbx2^tm1Cim^/J) [23] were obtained from the Jackson Laboratory (Bar Harbor, ME). To obtain fetuses carrying a homozygous null mutation (*Cbx2*^(−/−)^), heterozygous mating pairs were set up and fetal gonads were collected on day 18.5 pc. Genotyping of *Cbx2*^(−/−)^ mice was conducted according to the Jackson Laboratory's genotyping protocol for Cbx2^tm1Cim^, version 1.1 using the following primer pairs: “Cbx2-fwd AAC CGG AAG AGA GGC AAG AG”; “Cbx2-rev CCT GAA GGA GCA ACA AGA AAG”; “Neo-rev AGG TGA GAT GAC AGG AGA TC”. Genomic tail DNA was extracted by using the DNeasy Blood and Tissue Kit (QIAGEN Sciences, Germantown, MD) following manufacturer's instructions. The PCR conditions to determine the genotype were as follows: 94 °C for 3 min; 38 cycles of 94 °C for 30 s, 58 °C for 1 min and 72 °C for 1 min; followed by 72 °C for 10 min, and maintained at 4 °C until gel electrophoresis (Bio-Rad Laboratories, Hercules, CA). The chromosomal sex of each fetus was analyzed in parallel using the primer pair “Sry-fwd CTG TGT AGG ATC TTC AAT CTC T” and “Sry-rev GTG GTG AGA GGC ACA AGT TGG C” specifically amplifying a genomic region within the Y chromosomal SRY gene.

### 3.2. Histology and Analysis of Meiotic Configurations in Cbx2^(−/−)^ Fetal Germ Cells

Fetal gonads were dissected from control and *Cbx2*^(−/−)^ fetuses on day 18 pc and immediately fixed in Bouin's solution (Sigma, St. Louis, MI). Following overnight fixation, the gonads were washed in PBS solution and processed for paraffin embedding and sectioning according to standard procedures. 5 μm serial sections were stained with H&E before microscopy and image acquisition. Fetal gonads recovered from fetuses on day 18.5 pc were also processed for the analysis of chromosome synapsis and meiotic recombination proteins on surface spread germ cells as described previously [70]. Briefly, gonads were dissected and transferred to a hypotonic solution containing 1% sodium citrate for 20 min, before spreading of meiotic configurations onto glass slides and fixation in a solution of 1% paraformaldehyde, 0.1% Triton X 100.

### 3.3. Immunochemistry

Unless otherwise specified, all primary antibody incubations were conducted overnight at 4 °C. The extend of meiotic chromosome synapsis in control and mutant germ cells was determined by immunochemical detection of the lateral elements of the synaptonemal complex protein SYCP3 using a 1:1000 dilution of a rabbit anti-SYCP3 antibody (abcam, Cambridge, MA). An anti-phosphohistone H2AX (Ser-139) antibody (Upstate, Charlottesville, VA) was used at a 1:500 dilution. The type of meiotic configuration present in control and mutant oocytes at the pachytene stage was analyzed by simultaneous staining of the central element of the synaptonemal complex with a rabbit anti-SYCP1 antibody (1:500) and a guinea pig anti-SYCP3 at a 1:250 dilution [71]. Antibodies directed against BRCA1 (goat polyclonal, Santa Cruz, CA) and ubH2A (mouse monoclonal, clone E6C5, Millipore, Billerica, MA) were used at a dilution of 1:400. The mouse monoclonal anti-Mlh1 (BD Pharmingen) and mouse polyclonal anti-Rad51 (Oncogene) antibodies were both used following an overnight incubation at 37 °C at a 1:50 dilution in combination with a rabbit anti-SYCP3 antibody (1:250). Immunodetection was performed using appropriate Alexa Fluor-conjugated secondary antibodies (Molecular Probes, Eugene, Oregon, USA) at a dilution of 1:1000 for 2 h at room temperature.

Following immunochemistry, slides were counterstained with 7 μL of antifading medium supplemented with DAPI (Vectashield; Vector Laboratories, Burlingame, CA). Epifluorescence analysis of meiotic chromosomes was conducted on a DMRX/E microscope (Leica Microsystems) using a 100× objective. Images were captured with a Leica DFC 350F camera using Openlab 3.1.7. software (PerkinElmer) and image processing was performed using Photoshop 2.0 (Adobe) for linear adjustments and cropping of fluorescent images. No gamma adjustments were made.

### 3.4. Fluorescence in situ Hybridization (FISH)

The position of the sex chromosomes in control and mutant germ cells was determined by Immuno-FISH analysis following immunochemistry on the same slide using a FITC-conjugated X and a Cy3-conjugated Y chromosome paint (STARFISH, Cambio) according to manufacturer's specifications. Briefly, surface spread interphase nuclei and metaphase chromosomes were denatured in 70% formamide (VWR International Ltd., Poole, UK) in 2 × SSC at 80 °C for 10 min and subsequently chilled in ice-cold 70% ethanol for 5 min. The X and Y chromosome-specific probes were denatured for 10 min at 85 °C, respectively, and incubated at 40 °C for 1 h. Overnight hybridization was carried out in a humidified chamber at 40 °C and stringency washes were conducted in a solution containing 50% formamide in 2 × SSC as previously described [64,72]. Co-localization of a fully synapsed bivalent with the signal provided by the X chromosome-specific probe was considered indicative of complete homologous pairing of the X bivalent in XX oocytes at the pachytene stage.

### 3.5. Statistical Analysis

Data presented as percentage values were analyzed by one-way analysis of variance (ANOVA) following arcsine transformation. Comparison of all pairs was conducted by the Tukey-Kramer HSD or Student's t-test using JMP Start Statistics (SAS Institute Inc., Cary, NC). Variation among individual replicates is indicated as the standard deviation (s.d.). Differences were considered significant when *P* < 0.05 and are indicated by different superscripts.

## 4. Conclusions

The Cbx2 protein is expressed in the human embryonic gonadal ridge at seven weeks of gestation, consistent with its prominent role in early gonadal growth and differentiation [73]. Importantly, lack of Cbx2 function results in male to female sex reversal [18,21]. However, our study revealed major differences in the sex reversal phenotype according to the genetic background in mice. The milder phenotype observed in the BALB/C model allowed for the identification of a critical role for Cbx2 in germ cell development. Our studies uncovered a previously unrecognized role for Cbx2 in maintaining the pre-meiotic arrest in male germ cells and suggest that germ cell-specific chromatin modifications and structure may be an important component to confer specificity to the otherwise pleiotropic effects of RA on gonadal differentiation. Importantly, our studies uncovered a critical role for Cbx2 in homologous chromosome synapsis and chromosome stability in the female germline. These results may have important implications to understand the mechanisms involved in previously reported cases of pediatric patients exhibiting male to female sex reversal [20].
